# Immuno-Isolating Dual Poly(ethylene glycol) Capsule Prevents Cancer Cells from Spreading Following Mouse Ovarian Tissue Auto-Transplantation

**DOI:** 10.20900/rmf20190006

**Published:** 2019-09-16

**Authors:** James R. Day, Anu David, Catherine Long, Grace G. Bushnell, Teresa K. Woodruff, Lonnie D. Shea, Ariella Shikanov

**Affiliations:** 1 Department of Biomedical Engineering, University of Michigan, Ann Arbor, MI 48109, USA; 2 Department of Obstetrics and Gynecology, Northwestern University, Evanston, IL 60611, USA; 3 Department of Obstetrics and Gynecology, University of Michigan, Ann Arbor, MI 48109, USA; 4 Department of Macromolecular Science and Engineering, University of Michigan, Ann Arbor, MI 48109, USA

**Keywords:** female cancer survivors, ovarian tissue auto-transplantation, immuno-isolation, poly(ethylene glycol)

## Abstract

For female cancer survivors, premature ovarian insufficiency (POI) is a common complication of anticancer treatments. Ovarian tissue cryopreservation before treatment, followed by auto-transplantation after remission is a promising option to restore fertility and ovarian endocrine function. However, auto-transplantation is associated with the risk of re-introducing malignant cells harbored in the stroma of the ovarian autograft. To mitigate this risk, we investigated in this pilot study whether an immuno-isolating dual-layered poly(ethylene glycol)(PEG) capsule can retain cancer cells, while supporting folliculogenesis. The dual PEG capsule loaded with 1000 4T1 cancer cells retained 100% of the encapsulated cells *in vitro* for 21 days of culture. However, a greater cell load of 10,000 cells/capsule led to capsule failure and cells’ release. To assess the ability of the capsule to retain cancer cells, prevent metastasis, and support folliculogenesis *in vivo* we co-encapsulated cancer cells with ovarian tissue in the dual PEG capsule and implanted subcutaneously in mice. Control mice implanted with 2000 non-encapsulated cancer cells had tumors formed within 14 days and metastasis to the lungs. In contrast, no tumor mass formation or metastasis to the lungs was observed in mice with the same number of cancer cells encapsulated in the capsule. Our findings suggest that the immuno-isolating capsule may prevent the escape of the malignant cells potentially harbored in ovarian allografts and, in the future, improve the safety of ovarian tissue auto-transplantation in female cancer survivors.

## INTRODUCTION

Premature ovarian insufficiency (POI) is a common complication of anticancer treatments, such as chemo- and radio-therapy, due to ovarian sensitivity to these treatments [1,2]. In recent years, the survival rate of children with cancer has increased to over 85% due to the development of modern anticancer therapies; however, these patients experience long-term health problems far after they’re cancer-free [3,4]. Female cancer survivors with POI suffer from sterility and a myriad of problems associated with ovarian hormone deficiencies, such as premature osteopenia, muscle wasting, and cardiovascular disease [5]. Fertility preservation options, such as oocyte and embryo cryopreservation, are available for post-pubertal adolescent girls and young women. However, these options are not available for prepubertal patients [6].

For prepubertal patients, an experimental option of cryopreservation of ovarian tissue before exposure to toxic treatments, and subsequent auto-implantation after remission would restore both fertility and ovarian endocrine function [7–17]. Ovarian tissue auto-transplantation has resulted in close to 100 births to date [18]. Importantly, ovarian tissue auto-transplantation is associated with a significant risk of re-introducing malignant cells harbored in the autologous transplant, particularly in the case of hematologic malignancies, which is common in children [14,19–21]. A few patient cases of ovarian auto-transplantation in Europe resulted in relapse and multiple experiments in mice further confirmed the risks associated with this approach [19–22]. A potential option for these patients that would minimize the risk of cancer re-introduction is allotransplantation of donor ovarian tissue [23–25], however this option would solely provide ovarian endocrine function restoration and not provide an option for biologic fertility preservation.

To date, there is no standard universal protocol to ensure the absence of malignant cells in ovarian autografts removed from the patient prior to anti-cancer treatments. Several groups attempted to identify and quantify cancerous cells present in ovarian tissue using histology or PCR [19,21,22]. However, challenges in identifying cancer cells harbored in the tissue prevent clinical translation of this approach, especially in the case of hematologic malignancies, such as acute lymphoblastic leukemia, acute myeloid leukemia, and non-Hodgkin’s lymphoma, where ovarian involvement can be high. For example, even when histological analysis showed no evidence of malignant cells, an increase in cancer biological markers was shown through PCR analysis [19,21,22]. It’s recommended that the use of molecular markers be used to detect malignant cells, because solely histology is not sufficient; however, not all cancers display specific genetic markers that can be detected via PCR making tissue characterization very difficult [26]. Additionally, there is no consensus as to which markers are most sufficient to detect malignant cells present in ovarian tissue, dependent on the strain of cancer [22], further complicating the classification of the ovarian tissue as safe.

We hypothesized that immuno-isolation may mitigate the risk of re-introducing malignant cells during ovarian tissue auto-transplantation. Immuno-isolation uses a semi-permeable membrane to encapsulate foreign allogeneic tissue and allow free diffusion of nutrients and metabolites, while preventing the infiltration of immune cells attenuating the immune response. Previously, we have shown that an immune-isolating multilayered poly(ethylene glycol)(PEG) capsule supports ovarian tissue *in vivo* and did not elicit a measurable inflammatory response [25,27]. The immuno-isolating capsule contains a degradable core that is conducive for ovarian tissue survival and a non-degradable shell, which acts as the immunoprotective barrier. We have demonstrated that allogeneic ovarian tissue does not elicit a host immune response when encapsulated in the Dual PEG capsule, and the capsule prevents lymphocytic infiltration, allowing for allogeneic transplantation of ovarian tissue. Because the immuno-isolating capsule prevents infiltration of immune cells from the host towards the allograft through the barrier, we hypothesized that the capsule will contain cancer cells harbored in the ovarian tissue and will prevent their escape. Thus, encapsulating ovarian autografts in the immuno-isolating capsule mitigates the risk of cancer relapse or metastasis. Here, we investigated the ability of the immune-isolating capsule to prevent spreading of the cancer cells *in vitro* and *in vivo*, preventing tumor mass formation and metastasis, while maintaining the function of ovarian tissue. Balb/c derived 4T1 breast cancer cells originate from an invasive cancer line and were chosen to investigate the effectiveness of the capsule to retain cancerous cells and detect metastasis in a scenario when cancer cells escape the capsule and invade the host. This work aims to mitigate the risk of ovarian tissue auto-transplantation and provide female cancer survivors experiencing POI with safer options to restore fertility and hormonal balance.

## MATERIALS AND METHODS

### Gel Preparation

Degradable poly(ethylene glycol) hydrogels (PEG-MT) were formed via Michael-type addition. To prepare degradable PEG-MT hydrogels for the core, 8-arm PEG-VS (40kDa, Jenkem Technology, Beijing, China) was cross-linked with a plasmin sensitive tri-functional peptide sequence (AcGCYK↓NSGCYK↓NSCG, MW 1525.69 g/mol, >90% Purity, CelTek, ↓ indicates the cleavage site of the peptide). The non-degradable shell for Dual PEG hydrogels were prepared using 4-arm PEG-VS 20 kDa, Jenkem Technology, Beijing, China) with Irgacure 2959 (Ciba, Basel, Switzerland, MW = 224.3) and 0.1% *N*-vinyl-2-pyrrolidone (Sigma-Aldrich, St. Louis, USA). The detailed protocol is described in Day *et al.* [25].

### Collection of Murine Donor Ovaries

Donor ovaries were collected from 6–8 days old BALB/c mice. The collected ovaries were transferred to Leibovitz L-15 media (Sigma-Aldrich, St. Louis, USA) and dissected open. The ovarian tissue was then transferred into maintenance media (α-MEM; Gibco, Langley, USA), kept at 37 °C and 5% CO_2_ until encapsulated.

### 4T1 Cell and Ovarian Tissue Encapsulation

BALB/c derived 4T1 cancer cells were encapsulated at a concentration of 250,000 cells/mL (1000 cells/gel) or 2.5 million cell/mL (10,000 cells/gel). For encapsulation in the PEG-MT, the cancer cells and/or ovarian tissue were transferred into a 4 μL droplet of the plasmin sensitive tri-functional peptide and PEG-VS precursors’ solution. The droplet was allowed to crosslink for 5 minutes and then was quenched in maintenance media. For Dual PEG hydrogel encapsulation, the tissue was first encapsulated in a 4 μL PEG-MT hydrogel and was then placed in the center of a 10 μL bead of PEG-VS precursor solution (5% w/v PEG-VS, .4% Irgacure 2959, 0.1% NVP) and exposed to UV light [16]. All constructs were imaged immediately after encapsulation of the cells and tissue to verify complete encapsulation.

### Subcutaneous Injection and Implantation

For controls, a cell suspension containing 2000 or 20,000 cancer cells was injected subcutaneously on the dorsal side of the anesthetized mice (BALB/c). For animals receiving the cancer cells/ovarian tissue encapsulated in Dual PEG, a small incision was made on the dorsal side of the anesthetized mice (BALB/c) and 2 capsules per mouse were placed subcutaneously containing either 1000 or 10,000 cancer cells in each capsule making each mouse receive a total of 2000 or 20,000 cancer cells to compare to controls. The skin was closed using 5/0 absorbable sutures. The mice received Carprofen for analgesia for at least 24 h after surgery or as needed. Mice were monitored for two weeks to one month and euthanized at the end of the experiment. Control mice were sacrificed at 14 days and mice receiving Dual PEG were sacrificed after one month. *N* = 5 for each group.

The IACUC guidelines for survival surgery in rodents and the IACUC Policy on Analgesic Use in Animals Undergoing Surgery were followed for all the procedures. Animal experiments for this work were performed in accordance with the protocol approved by the Institutional Animal Care and Use Committee (IACUC) at the University of Michigan (PRO00007716) in May 2017.

### IVIS Imaging

Bioluminescent imaging via the IVIS (*In vivo* Imaging System) was utilized to track cell growth during the implantation period. Implanted 4T1 cells were labeled with firefly luciferin. Briefly, on the day of implantation and every 7 days during the time course of the study, 150 μL of 63 mM substrate (Xenolight D-Luciferin-K+ Salt Bioluminscent substrate, PerkinElmer, Waltham, USA) was injected intraperitoneally. After 10 min of incubation, mice were imaged. Similarly, to assess metastasis, collected organs and tumors on day of sacrifice were imaged by adding 28 μL of 63 mM substrate per mL of culture media. Images were processed and analyzed using LivingImage software (version 4.7.2).

### Tumor, Gel, and Organ Collection

At the time of sacrifice, tumors or gels were collected, imaged via IVIS, and fixed in Bouin’s fixative. Additionally, the brain, liver, lungs, spleen, blood, and ovaries were collected, imaged via IVIS, and fixed in Bouin’s fixative.

### Histological Analysis of Retrieved Capsules and the Encapsulated Ovarian Tissue

Following sacrifice, the immuno-isolating devices were retrieved from mice, fixed in Bouin’s fixative at 4 °C overnight, transferred and stored in 70% ethanol at 4 °C. After processing, samples were embedded in paraffin, serially sectioned at 5 μm thickness, and stained with hematoxylin and eosin.

### Statistics

A Pearson’s (*N* − 1) Chi-squared test was performed. Calculated values were performed using R, and all *p*-values were evaluated against a significance level (α) of 0.05. To test significance in change in radiance over time, a Welch’s *t*-test was used with *p* < 0.05 determined as significant.

## RESULTS

### Encapsulation of Cancer Cells in Dual PEG Prevents Cancer Cell Escape in Culture

**Figure 1.**
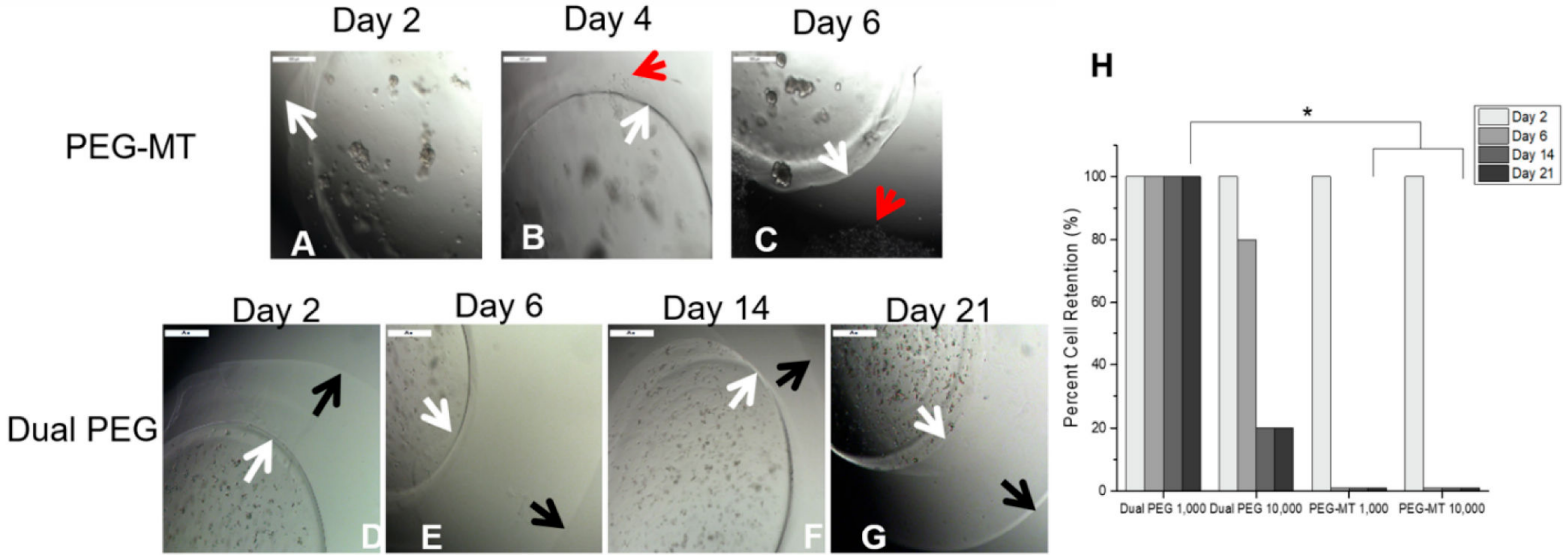
*In vitro* encapsulation of 1000 4T1 cells in (**A**–**C**) PEG-MT and (**D–G**) Dual PEG. (**H**) Percentage of hydrogels (*n* = 5) for each group that retained encapsulated cancer cells through 21 days of culture. White arrow indicates border of PEG-MT hydrogel, red arrow indicates escaped cancer cells, and black arrow indicates border of non-degradable shell in Dual PEG. * indicates statistical significance (*p* < 0.05).

First, we evaluated the ability of the immuno-isolating capsule to retain cancer cells and prevent their migration and/or proliferation in the respective capsule. To determine whether cancer cells escaped from the gels, the cell-containing capsules were placed in non-tissue treated well plates and imaged every other day. One thousand and 10,000 cells per gel were encapsulated in both PEG-MT, a degradable capsule, and Dual PEG, a capsule containing a degradable core and non-degradable shell. For Dual PEG, cells were only encapsulated in the degradable core component of the capsule, which would mimic the clinical scenario where only the ovarian tissue surrounded by a degradable hydrogel contains cancer cells. Cells encapsulated in PEG-MT escaped the capsule by Day 6 in 100% of the gels for both the 1000 and 10,000 cell/gel groups (*n* = 5)(Figure 1A–C,H).

With the inclusion of the non-degradable shell in the case of Dual PEG, cell escape out of the capsule significantly decreased (*p* < 0.05). Dual PEG gels with 1000 cells encapsulated in its core showed 0% escape through 21 days culture (Figure 1D–H). When the cell concentration was increased and 10,000 cells were encapsulated in PEG-Dual, cells did escape with 80% of gels showing some extent of cell escape by day 21. However, the kinetics of cell escape was attenuated compared to PEG-MT, as at day 2, 100% of Dual PEG gels containing 10,000 retained the cells in the core and at day 6, 80% showed complete retention (Figure 1H). This demonstrated that the inclusion of the non-degradable shell greatly hinders cell migration and proliferation out of the gel, leading to the retention of cells in the capsule through 21 days *in vitro* culture.

### Encapsulation of Cancer Cells in Dual PEG Prevents Spreading and Metastasis *in Vivo*

**Figure 2.**
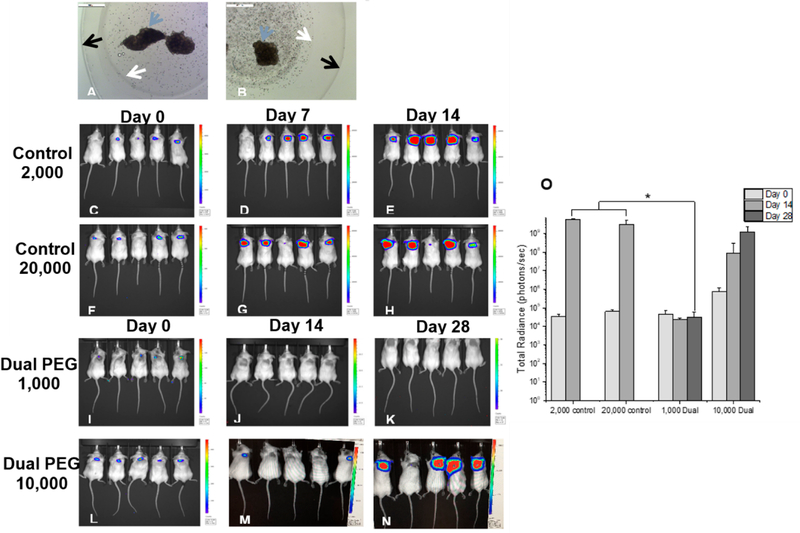
Microscopic image of (**A**) 1000 and (**B**) 10,000 4T1 cells co-encapsulated with BALB/c ovarian tissue in Dual PEG. Magnification 5×. Bioluminescent imaging of mice receiving (**C–E**) 2000 non-encapsulated 4T1 cells, (**F–H**) 20,000 non-encapsulated 4T1 cells, (**I–K**) 2 Dual PEG capsules both encapsulating 1000 4T1 cells and ovarian tissue and (**L–N**) 2 Dual PEG capsules both encapsulating 10,000 4T1 cells and ovarian tissue. (**O**) Average radiance of mice receiving control non-encapsulated cells or cells encapsulated in Dual PEG (*n* = 5 per group). White arrow indicates border of degradable PEG-PD core, black arrow indicates border of non-degradable shell in Dual PEG, and blue arrow indicates ovarian tissue. * indicates statistical significance (*p* < 0.05).

**Figure 3.**
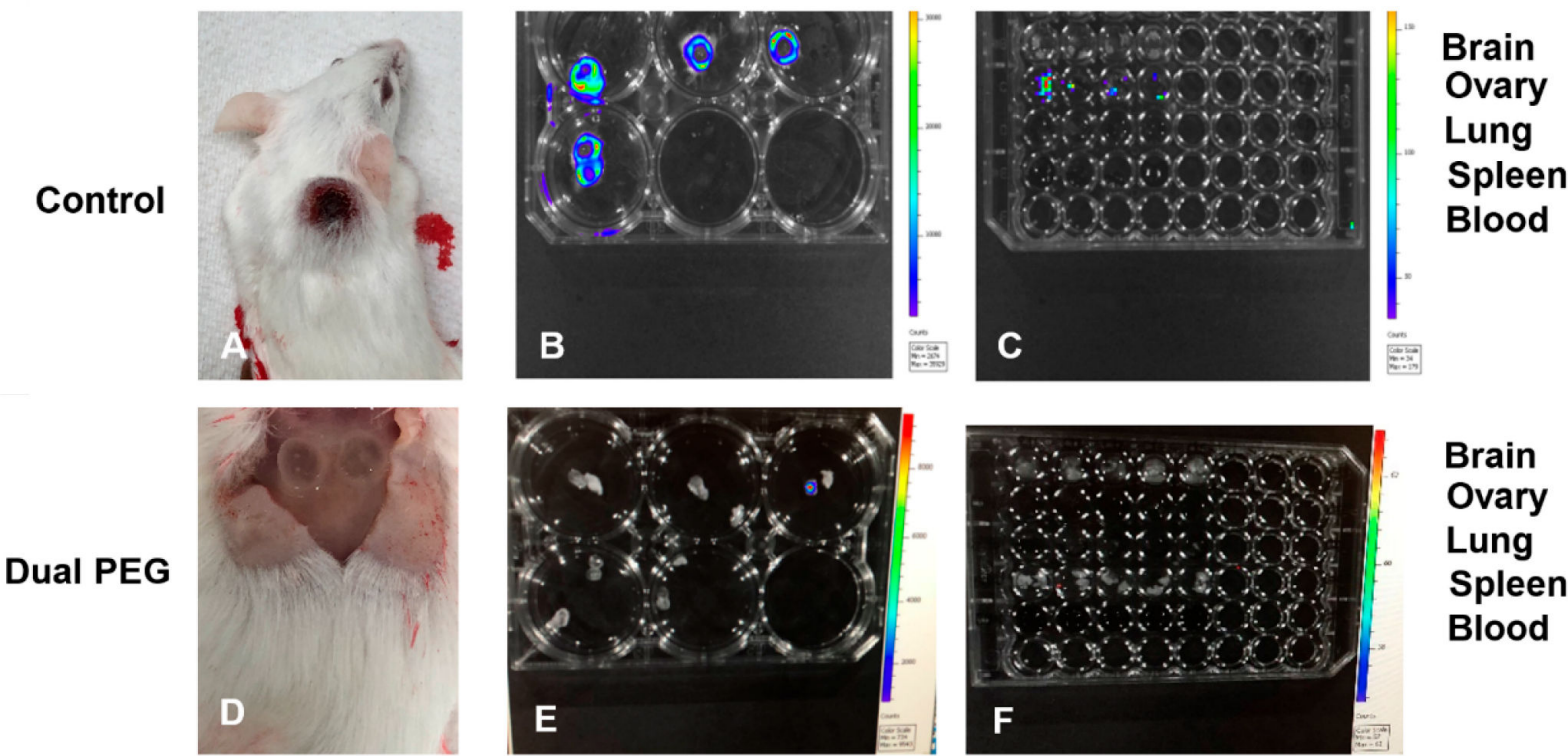
(**A**) Macroscopic image of mice receiving non-encapsulated 4T1 cells and ovarian tissue. Bioluminescent imaging of (**B**) resected tumors and (**C**) organs following sacrifice 14 days after injection. Organs removed include brain, ovary, lung, blood, and spleen. (**D**) Macroscopic image of mice receiving Dual PEG containing 1000 4T1 cells and ovarian tissue. Bioluminescent imaging of (**E**) resected capsules and (**F**) organs following sacrifice 28 days after implantation. White circle indicates location of hydrogels.

To evaluate the Dual PEG capsule’s ability to retain cancerous cells *in vivo*, cells were co-encapsulated with mouse ovarian tissue and implanted subcutaneously in a BALB/c mouse model. The cancer cell line, mouse ovarian tissue, and recipient mouse strain were all the same to negate any possible immune response. Either, 1000 or 10,000 cells, were encapsulated in Dual PEG with 6–8 day old ovarian tissue (Figure 2A,B). Each mouse (*n* = 5 per group) received two identical capsules. For positive controls, a cell suspension containing either 2000 or 20,000 4T1 cells was injected subcutaneously. When 2000 cells were injected, proliferation was exhibited in 5/5 mice with radiance increasing from 3.56 × 10^4^ to 6.02 × 10^9^ photons/sec from day 0 to 14 (Figure 2C–E,O). By Day 14, all mice had to be sacrificed as a tumor mass had formed on the dorsal side and ulceration started to occur (Figure 3A). In the mice receiving 20,000 non-encapsulated cancer cells, proliferation and tumor mass formation was observed in 4/5 mice (Figure 2F–H,O). Similar to the 2000 cell group, the mice exhibited ulceration by day 14 and had to be sacrificed. The cells proliferated as shown by the significant increase of radiance from day 0 to day 14 (Figure 2O). To confirm the extracted tumor masses were in fact the labeled 4T1 cells, bioluminescence imaging was used. All tumors exhibited bioluminescence indicating the mass was cancerous and derived from the injected 4T1 cells (Figure 3B).

When 1000 cells were encapsulated in Dual PEG and two capsules were implanted per mouse (total of 2000 cells per mouse), 0 out of 5 mice exhibited a tumor mass formation through 28 days as shown through a non-significant change in radiance from day 0 to day 28 (Figure 2I–K,O). From IVIS imaging, it appeared that all cancer cells were retained within the capsule. To confirm, when hydrogels were extracted, the capsules as well as the surrounding tissue were imaged via bioluminescence and only the inner core of the hydrogels showed luminescent signal (Figure 3E). Additionally, upon macroscopic evaluation, the hydrogels containing cancer cells and ovarian tissue looked exactly as they did when implanted on day 0 and no tumor mass was present (Figure 3D). For the mice receiving 10,000 cancer cells co-encapsulated with ovarian tissue in Dual PEG (2 devices, 20,000 cancer cells total per mouse), 4/5 mice demonstrated tumor formation as shown through a significant increase in radiance over the 28 day implantation period (Figure 2L–O). Upon extraction, a tumor mass localized to the hydrogels was evident as the hydrogel appeared cloudy in appearance (Supplementary Figure S1). However, although the 10,000 cancer cells proliferated over the 28 day implantation period, the cells remained localized to the hydrogel, as the hydrogel showed a high level of radiance, indicating the cancer cells were present within the capsule, itself (Supplementary Figure S1).

To assess metastasis, organs such as the brain, ovaries, lungs, spleen, blood, and liver were explanted and examined for the presence of cancerous cells through bioluminescent imaging. In the 2000 and 20,000 cell control groups, radiance was exhibited in all the lungs, indicating cancerous cells metastasize to the lungs, which is expected for this cell line (Figure 3C). When 4T1 cells were encapsulated in Dual PEG, none of the extracted organs in any animal exhibited bioluminescence indicating the encapsulation prevented metastasis of cancerous cells to the lungs (Figure 3F) contrary to the non-encapsulated control.

**Figure 4.**
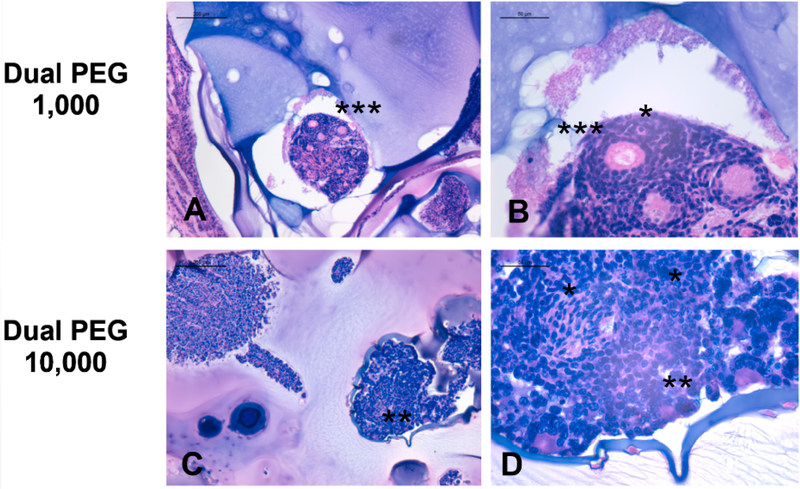
Histological image of ovarian tissue encapsulated in Dual PEG with (**A**,**B**) 1000 4T1 cells and (**C**,**D**) 10,000 4T1 cells. Scale bars: 200 μm (**A**,**C**) and 50 μm (**B**,**D**). * indicates primordial follicles, ** indicates primary follicles, and *** indicates secondary follicles.

Lastly, to assess the ability of ovarian tissue to survive and develop in the presence of cancer cells, histological analysis was performed after explantation of the Dual PEG capsules to investigate the degree of folliculogenesis. Healthy, developing primary and secondary follicles were seen in ovarian tissue co-encapsulated with 1000 and 10,000 cancerous cells (Figure 4) demonstrating folliculogenesis was supported. Interestingly, a higher number of follicles were observed in the ovarian tissue encapsulated with 1000 cancer cells compared to 10,000 cells, which could be due to damage to ovarian tissue by the cancerous cells [28]. Multiple primordial follicles are present in capsules with 1000 and 10,000 cancer cells (Figure 4B,D).

## DISCUSSION

Cryopreservation and subsequent auto-transplantation of a patient’s own ovarian tissue would be an optimal option for young female cancer survivors, as it gives patients the option to have biological children long after treatment and restores their natural hormone function [5–10]. However, auto-transplantation presents the patients with a risk of re-introducing malignant cells, as the ovarian tissue was resected before treatment leading to the possibility of malignant cells being stored in the stroma of the tissue. In hematological cases, which is a high percentage of childhood cancers, this risk and ovarian involvement is very high [14,19–22]. Previous studies have been conducted to identify cancerous cells present in resected ovarian tissue from cancer patients. Due to discrepancies in characterization methodology, molecular marker criteria, and tissue heterogeneity, no universal protocol has been established to characterize ovarian tissue as safe [19–22] creating a risk of re-seeding malignant cells for female cancer survivors following ovarian tissue auto-transplantation.

This work describes a method to mitigate this risk by retaining cancerous cells in a capsule which is also conducive for ovarian tissue survival and development. Previously, we developed a multilayered immuno-isolating capsule, Dual PEG, which contains a degradable core that is conducive for ovarian tissue development and a non-degradable shell that acts as an immunoprotective barrier [25]. We demonstrated that when encapsulating allogeneic ovarian tissue and using an allogeneic mouse model, larger immune cells such as cytotoxic T cells are unable to penetrate the capsule. If cells are unable to penetrate the capsule from the outside immune environment, we hypothesized the Dual PEG capsule could retain encapsulated cancerous cells present in ovarian tissue and not let cells escape.

First, we tested Dual PEG’s ability to retain cancerous cells *in vitro* in comparison to a degradable PEG-MT capsule. We demonstrated that by adding the non-degradable photopolymerized PEG layer, the ability of the capsule to retain cancer cells significantly increases. Through 21 days of culture, we showed 100% effectiveness of cancer cell retention when 1000 cells were encapsulated in Dual PEG, compared to 0% cancer cell retention through 2 days when encapsulated in PEG-MT. This difference is due to the nature of the crosslinks in the respective capsules. In PEG-MT, the crosslinks are formed via a protease-sensitive peptide, so when the cancer cells grow and proliferate, they secrete proteases, which break down the bonds of the hydrogel. This opens up the pores and network of the hydrogel, allowing the cells to escape from the gel. The photopolymerized layer is not susceptible to proteolytic or hydrolytic degradation as the crosslinks are formed via free radicals reacting with double bonds. We observed that when the amount of cancer cells was increased to 10,000 per Dual PEG capsule, cancer cells did escape in a portion of the gels. This may be due to incomplete encapsulation of the cancer cell-containing core or the core being broken down and swelling past the outer shell’s capabilities. However, this cell concentration of 2.5 million cells/mL is far above what would be present in a clinical situation and was used to test the upper limits of Dual PEG.

Next, we tested the ability of Dual PEG to retain cancer cells and prevent tumor mass formation and metastasis *in vivo* in a syngeneic mouse model. Previous studies have suspended 100–200 cancerous cells in a fibrin or alginate matrix, and when implanted into mice, did not reintroduce leukemic cell contamination [29,30]. Here, we encapsulated BALB/c mouse ovarian tissue with BALB/c derived 4T1 cancer cells (1000 or 10,000 cells per capsule) in Dual PEG and implanted two devices subcutaneously in BALB/c mice. A syngeneic model was used to mimic auto-transplantation of cancer cell-containing ovarian tissue. We compared the controls of injections containing cell suspensions of 2000 or 20,000 4T1 cells to match the total amount of encapsulated cells in the mice receiving Dual PEG. When non-encapsulated cancer cells were injected subcutaneously, we observed rapid cell proliferation via luminescent imaging as well as tumor mass formation. By Day 14, we had to sacrifice all control mice as the tumors resulted in ulceration. Additionally, luminescent signal was observed in the lungs, indicating the 4T1 cells metastasized to the lungs, which is expected for this cancer cell line. When encapsulating 1000 cancer cells (250,000 cells/mL) in Dual PEG, we observed no change in luminescent signal in all mice through 28 days indicating the absence of tumor mass formation. Additionally, there were no cancer cells present in any of the surrounding tissue or organs of interest, indicating cancer cells were retained within the capsule and metastasis had not occurred. All observed luminescent signal was concentrated in the capsule, further confirming that all cancer cells present were retained in the capsule throughout the whole implantation period. When the amount of cell in each capsule was increased in 10,000 (2.5 million cells/mL), an increase in luminescent signal was observed over the 28 day implantation period indicating cell proliferation and tumor mass formation was taking place; however, all the cells were maintained in the gel as indicated by the lack of cells in surrounding tissues and susceptible organs. Macroscopically, we were able to see the tumor mass within the gel itself and upon IVIS imaging, the hydrogel was shown to be the only entity that contained cancer cells. At a high cell density of 10,000 cells/capsule, the Dual PEG gel is able to retain those cells and prevent metastasis, unlike the non-encapsulated control. When the capsules were explanted and histological analysis was performed, we observed the presence of healthy primary and secondary follicles. This is vital to a successful encapsulation strategy for auto-transplantation, as this shows the tissue is capable of surviving within the capsule, potentially restoring fertility and ovarian endocrine function.

## CONCLUSIONS

This work demonstrated the capability of using an immuno-isolating capsule to retain cancer cells that may be present in an ovarian tissue autograft. Previously, we have shown that Dual PEG can support ovarian tissue survival and development, leading to the restoration of ovarian endocrine function. We have also demonstrated the capsule’s ability to protect encapsulated ovarian tissue from an immune response. By using this capsule to encapsulate a patient’s ovarian autograft, the risk of re-introducing malignant cells present in that tissue is mitigated. Given there is no protocol by which clinical investigators can be absolutely certain ovarian autografts do not contain cancerous cells, girls that had hematologic malignancies, and showed zero to a low concentration (250,000 cells/mL) of cancerous cells in their ovarian biopsy, could potentially use of an immuno-isolating capsule to decrease the risk of cancer re-introduction. Through this work, the option of auto-transplantation is potentially safer, which, although experimental, is the only option for pre-pubescent female cancer survivors to retain, both, future fertility and ovarian endocrine function. Future work will be done to assess the ability of encapsulated mouse ovarian tissue to produce viable eggs once encapsulated in the Dual PEG capsule. To this end, developing new methods to create a capsule using alternatives to photopolymerization may be necessary. Limitations of this study include the use of mouse ovarian tissue and a short *in vivo* transplantation period. For this reason, future studies encapsulating human ovarian tissue will be conducted to investigate the ability of the PEG capsules and other biocompatible materials to accommodate the large volumetric expansion of human follicles during development and the ability for capsule to support ovulation and corpus luteum formation.

## Supplementary Material

Supplemental

## ABBREVIATIONS

POI: premature ovarian insufficiency
PEG: poly(ethylene glycol)
PCR: polymerase chain reaction
IVIS: *in vivo* imaging system
