# Supplementary material for: Immuno-Isolating Dual Poly(ethylene glycol) Capsule Prevents Cancer Cells from Spreading Following Mouse Ovarian Tissue Auto-Transplantation
Source: Regen Med Front. Author manuscript; Available in PMC 2021 May 6. (PMC8101948; doi:10.20900/rmf20190006)
Supplement: Supplemental [file NIHMS1065774-supplement-Supplemental.pdf]

Supplementary Materials

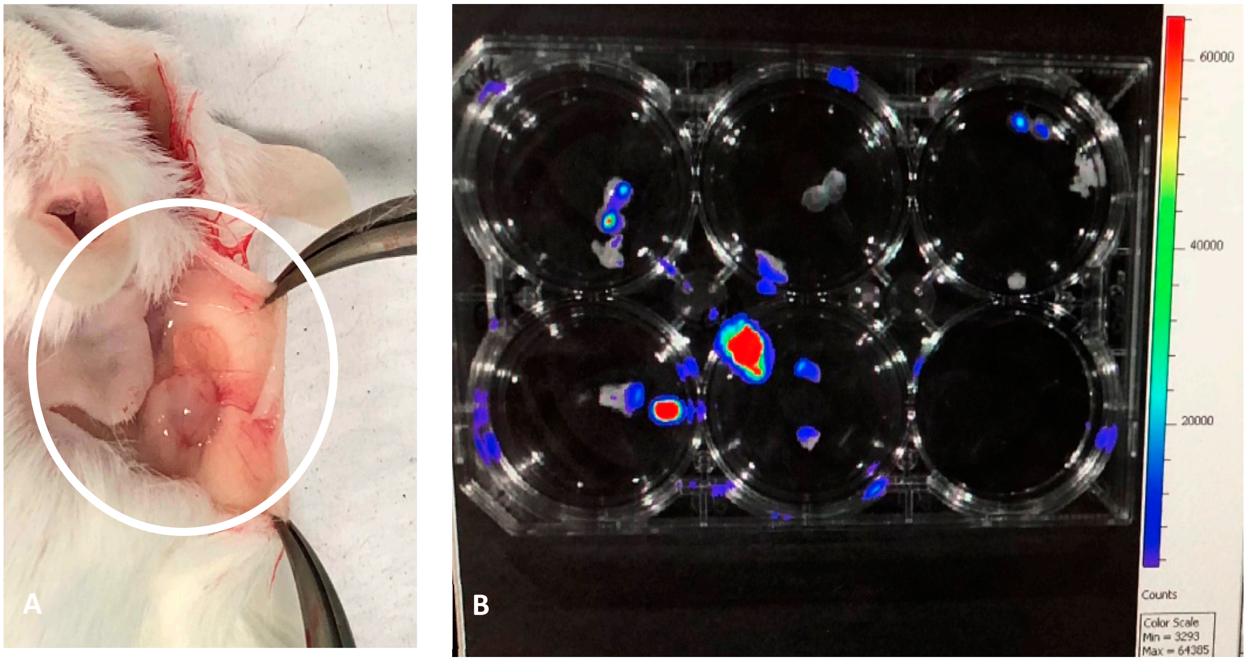

Supplementary Figure S1. (**A**) Macroscopic image of mice receiving Dual PEG containing 10,000 4T1 cells and ovarian tissue. (**B**) Bioluminescent imaging of resected capsules containing 10,000 4T1 cells following sacrifice 28 days after implantation. White circle indicates the location of the capsules and tumor mass.
